# Dental and Skeletal Effects of Herbst Appliance, Forsus Fatigue Resistance Device, and Class II Elastics—A Systematic Review and Meta-Analysis

**DOI:** 10.3390/jcm11236995

**Published:** 2022-11-26

**Authors:** Stefanos Matthaios, Apostolos I. Tsolakis, Anna-Bettina Haidich, Ioannis Galanis, Ioannis A. Tsolakis

**Affiliations:** 1 Department of Orthodontics, School of Dental Medicine, Case Western Reserve University, Cleveland, OH 44106, USA; 2 Department of Orthodontics, School of Dentistry, National and Kapodistrian University of Athens, 11527 Athens, Greece; 3 Department of Hygiene, Social-Preventive Medicine & Medical Statistics, Medical School, Aristotle University of Thessaloniki, 54124 Thessaloniki, Greece; 4 2nd Propaedeutic Department of Surgery, Medical School, Aristotle University of Thessaloniki, 54124 Thessaloniki, Greece; 5 Department of Orthodontics, School of Dentistry, Aristotle University of Thessaloniki, 54124 Thessaloniki, Greece

**Keywords:** class II malocclusion, herbst appliance, forsus fatigue resistance device, class II elastics, functional appliances, systematic review, meta-analysis

## Abstract

**Background**: Our study aimed to systematically summarize the dentoskeletal effects of Herbst appliance; Forsus fatigue resistance device; and Class II elastics in adolescent Class II malocclusion. **Methods**: Five databases; unpublished literature; and reference lists were last searched in August 2022. Randomized clinical trials and observational studies of at least 10 Class II growing patients that assessed dentoskeletal effects through cephalometric/CBCT superimpositions were eligible. The included studies quality was assessed with the RoB 2 and ROBINS-I tools. A random-effects model meta-analysis was performed. Heterogeneity was explored with subgroup and sensitivity analyses. **Results**: Among nine studies (298 patients); two-to-three studies were included in each meta-analysis. Less post-treatment upper incisor retroclination (<2) and no overbite; overjet; SNA; SNB; and lower incisor inclination differences were found between Herbst/Forsus and Class II elastics. No differences in maxilla; condyle; glenoid fossa; and most mandibular changes were found between Herbst and Class II elastics; except for a greater 1.5 mm increase in mandibular length and right mandibular ramus height (1.6 mm) with Herbst. **Conclusions**: Herbst and Class II elastics corrected the molar relationship; but Herbst moved the lower molars more mesially. Apart from an additional mandibular length increase; no other dental and anteroposterior skeletal difference was found. Forsus was more effective in molar correction; overjet reduction; and upper incisor control than Class II elastics. Trial registration number OSF: 10.17605/OSF.IO/8TK3R.

## 1. Introduction

One of the most common orthodontic problems is Class II malocclusion which is defined as the distal position of the mandibular first molar relative to the maxillary first molar according to Angle [1,2]. It presents a mean global distribution of 19.56% in permanent dentition [3] and a 12 to 32% range in Caucasians [4,5,6,7], while mandibular retrognathia, one of its major causes, seems to be influenced by the diet and function of the surrounding soft tissue [8,9,10,11,12].

Functional appliances force patients to hold their mandible forward causing the elevator muscles to stretch [13]. They can lead to Class II malocclusion correction by potentially increasing the mandibular length through condylar growth and glenoid fossa remodeling, restraining the maxilla and the upper teeth, and moving the lower teeth forward [13]. Accordingly, they can accelerate mandibular growth in growing patients, usually having a minor effect on the final mandibular size [13,14].

Herbst appliance is one of the most well-documented rigid fixed functional appliances [15] and is widely used for Class II malocclusion treatment [15]. It functions as an artificial joint between the jaws [15,16,17,18] by protruding the mandible forward and causing the “bite jumping” [15]. It also allows the patient to perform small lateral movements besides opening movements. The mean Herbst active treatment time is usually 6 to 8 months [15] while there seems to have stable beneficial dentoskeletal effects on Class II malocclusion correction [6].

Forsus Fatigue Resistance Device is a semi-rigid fixed functional appliance made from nickel-titanium coil springs. It is assembled chairside and is attached to an upper molar headgear tube and a lower heavy archwire at the mandibular canine bracket. It passively maintains the mandible forward, while allowing a limited mandibular movement [13]. The mean Forsus active treatment time is 6.2 months while there seems to have mainly dentoalveolar effects on Class II malocclusion correction [19].

Class II malocclusion can also be corrected by intermaxillary elastics that are attached to the brackets of the lower molars and the upper canines. Class II elastics can effectively move the lower teeth forward and the upper teeth backward [13], but they require patient compliance. Their mean active treatment time is 8.5 months, and they seem to have similar beneficial dentoalveolar effects to those of functional appliances [20], but can also have skeletal effects [21,22], while additional condylar remodeling has been shown in young monkeys [23]. On the other hand, in case of non-compliance, treatment time can be increased, and the overall results can be compromised.

While there are systematic reviews and meta-analyses [6,19,20] that investigate each appliance separately, no study has provided adequate evidence regarding their effectiveness on Class II malocclusion correction. More specifically, there were untreated controls in the included trials of these studies [6,19,20] and thus no inferences can be made about the comparative effectiveness of the appliances. Moreover, there is no systematic review and meta-analysis to include three-dimensional post-treatment differences between the appliances. This study aimed to identify, appraise, and summarize the evidence on the dentoskeletal effects of the Herbst appliance and Forsus Fatigue Resistance Device, compared to Class II elastics in the treatment of Class II growing patients.

## 2. Materials and Methods

### 2.1. Protocol and Registration

The protocol was registered on Open Science Forum Database following the PRISMA-P guidelines [24] (Protocol: 10.17605/OSF.IO/8TK3R, https://osf.io/pb7c4, accessed on 16 October 2022).

### 2.2. Eligibility Criteria

The following selection criteria were applied:

- Population: At least 10 Class II malocclusion-growing patients with all their permanent teeth present, except the third molars.
- Interventions/Comparators: Herbst or Forsus (non-TAD supported)/Class II elastics.
- Outcomes: Dentoskeletal changes measured by Cephalometric or CBCT analysis
- Study design: Randomized clinical trials and non-randomized trials.

### 2.3. Information Sources

A literature search without any language restriction was carried out independently and in duplicate by two authors (S.M., I.A.T.) in the following electronic databases: Medline database (via PubMed), Embase (via Ovid), Scopus, CENTRAL, and the Cochrane Oral Health Group’s Trial Register. Google Scholar, reference lists of the included studies, and conference proceedings and abstracts were also screened. The Polyglot Search Translator [25] of the Systematic Review Accelerator (SRA) website [26] was used to adapt each PubMed search term to each one of the other databases. The search strategy for each database has been evaluated against the PRESS checklist [27] and is presented in Appendix A.

### 2.4. Selection Process

All the retrieved studies were imported into the SRA [26], duplicates were removed using the Deduplicator option [26], and the advanced search of the Screenatron option [26] was used due to a large number of retrieved studies (Appendix A). Subsequently, studies were selected independently and in duplicate by two authors (S.M., I.A.T.), while any inconsistencies were resolved by discussion with a third author (A.I.T). The two authors (S.M., I.A.T.) were not blinded regarding the studies’ authors, institutions, or research findings. After identifying potentially relevant studies by title, abstracts were read, and non-eligible studies were eliminated. Then, a Google Scholar and a hand search of the eligible studies’ references were performed to potentially find additional articles not previously found. Finally, after reading the articles in full, a choice was made according to the eligibility criteria.

### 2.5. Data Collection

Two authors (S.M., I.A.T.) performed data extraction independently and in duplicate, and any discrepancies were resolved by consensus. All compatible results with each outcome domain were sought for all measures.

### 2.6. Data Items

The information that was extracted included: Trial characteristics, patient baseline characteristics, intervention characteristics, method of outcome assessment, outcomes, outcome results, and risk of bias assessment

### 2.7. Study Risk of Bias Assessment

The Revised Cochrane risk-of-bias tool for randomized trials (RoB 2) [28] and the ROBINS-I tool of Cochrane for non-randomized studies of interventions [29] were used to assess the quality of the included studies. Two authors assessed the articles individually and then compared their findings. Any disagreements were resolved by discussion with a third author (A.I.T.).

Regarding the randomized clinical trials, seven bias domains were assessed: bias arising from the randomization process, bias due to deviations from the intended interventions, bias due to missing outcome data, bias in the measurement of the outcome, and bias in the selection of the reported result. A judgment of ‘Low risk of bias’, ‘Some concerns’, or ‘High risk of bias’ was made for each domain, while an overall judgment was assessed based on the following:

a. Low risk of bias: if all the domains of the study were at low risk of bias.
b. Some concerns: if there were some concerns for at least one domain, but not a high risk of bias for any other domain.
c. High risk of bias: if at least one domain was at high risk of bias or multiple domains raised some concerns.

Regarding the non-randomized trials seven bias domains were assessed: bias due to confounding, bias in the selection of participants into the study, bias in the classification of interventions, bias due to deviations from the intended interventions, bias due to missing data, bias in the measurement of the outcomes, and bias in the selection of the reported results. A judgment of ‘Low risk of bias’, ‘Moderate risk of bias’, ‘Serious risk of bias’, and ‘Critical risk of bias’ was made for each domain, and an overall judgment was assessed based on the following:

a. Low risk of bias: if all domains of the study were at low risk of bias.
b. Moderate risk of bias: if all domains of the study were at low or moderate risk of bias.
c. Serious risk of bias: if at least one domain of the study was at serious risk of bias, but not at critical risk of bias in any other domain.
d. Critical risk of bias: if at least one domain of the study was at critical risk of bias.

### 2.8. Effect Measures

The mean and the standard deviation of the post-treatment differences between Herbst or Forsus and Class II elastics were used for the following outcomes: Overjet, overbite, upper and lower incisor inclination/position, molar relationship, molar inclination/position, maxillary and mandibular changes, jaw relationship, condylar changes.

### 2.9. Synthesis Methods

A meta-analysis was performed using a random-effects model for the following outcomes:

a. Overjet
b. Overbite
c. Upper incisor inclination
d. Lower incisor inclination
e. Maxillary changes
  - SNA angle
  - A point (AP change)
  - ANS point (AP change)
f. Mandibular changes
  - SNB angle
  - B point (AP change)
  - Pg point (AP change)
  - Right and left gonial angle (Co-Go-Me)
  - Right and left mandibular length (Co-Gn)
  - Right and left mandibular corpus (Go-Gn)
  - Right and left mandibular ramus height (Co-Go’)
g. Condylar and glenoid fossa changes
  - Right and left Co points (AP change)
  - Right and left anterior glenoid fossa (AP change)
  - Right and left posterior glenoid fossa (AP change).

Heterogeneity was evaluated on a clinical level, by performing subgroup analysis for the types of intervention (Herbst/Forsus) and on a statistical level, by assessing the x^2^ test and the I^2^ statistic. In the case of high heterogeneity levels (x^2^: *p*-value < 0.1 and I^2^ > 50%), a sensitivity analysis was performed, including only studies with a low overall risk of bias. All the analyses were performed with RevMan 5.4 software [30]. In the case of multiple groups in one study, the common group was split into two equal groups [31].

A narrative synthesis of the results was also used for all the outcomes. A table was also used to summarize the main findings of each study. The mean post-treatment difference and the standard deviation of the mean difference between Herbst or Forsus and Class II elastics were used.

### 2.10. Certainty Assessment

The evidence quality for all the outcomes was assessed using the GRADE reporting system criteria [32] by two authors (S.M., I.A.T.) who gave independently a final grade for the quality of evidence for each outcome. Any disagreements were resolved by consensus.

## 3. Results

### 3.1. Study Selection

**Figure 1:**
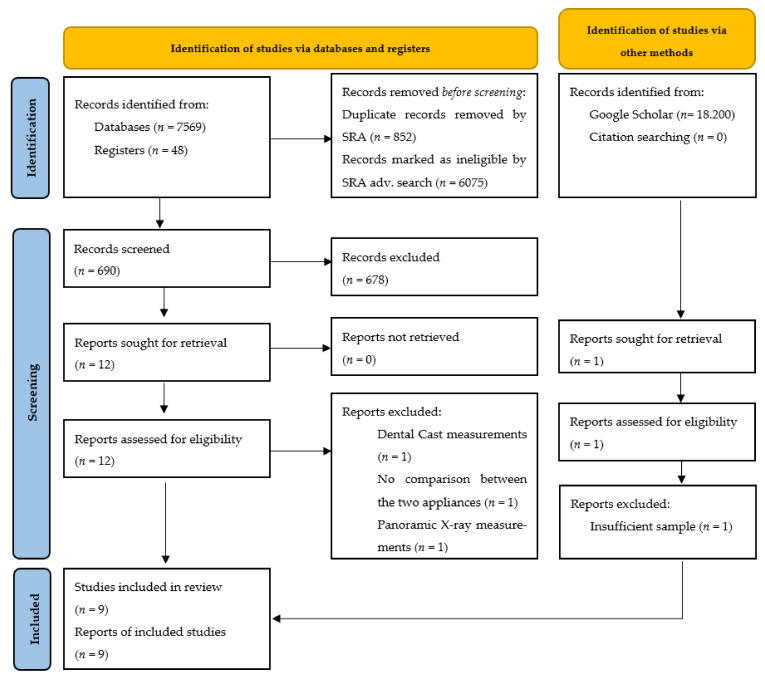
PRISMA 2020 flow diagram for new systematic reviews which included searches of databases, registers, and other sources [33].

Our search resulted in 7617 articles. After duplicate and irrelevant article exclusion using the SRA [26], the title and abstract of the remaining articles and the trials registered on ClinicalTrials.gov were screened, and 12 relevant articles were read in full. Finally, nine of them were selected based on our eligibility criteria. No additional eligible studies were found in the Google Scholar search or the included studies’ references search. The selection process is summarized below using the PRISMA 2020 flow diagram [33] (Figure 1).

The study of Griblasky et al. [34] was excluded due to the use of dental casts for the outcome measurements, while the study of Yin et al. [35] which compared the effects of Forsus and Class II elastics with the Carriere Distalizer appliance, had no direct comparison between the first two appliances. Finally, Çoban et al. [36] used panoramic X-rays instead of cephalometric radiographs or CBCT scans to evaluate external apical root resorption and Baldari et al. [37] had a smaller sample (*n* = 8) than our criterion of at least 10 patients in a study.

### 3.2. Study Characteristics

Among the nine included studies there were eight observational studies [38,39,40,41,42,43,44,45] and one randomized clinical trial [46]. There were four prospective [38,39,43,44] and three retrospective [40,42,45] observational studies comparing Herbst to Class II elastics. Additionally, there was one randomized clinical trial [46] and one retrospective observational study [41] comparing Forsus to Class II elastics. Their findings are summarized in Table 1.

### 3.3. Risk of Bias in Studies

The randomized clinical trial of Aras and Pasaoglu [46] was judged to present an overall low risk of bias and is presented in Table 2.

Most observational studies also presented an overall low risk of bias [38,39,40,42,43,44,45]. In the study of Jones et al. [41], patients treated with Forsus could have used elastics before or after its assignment while the cephalometric radiographs were taken pre-treatment and post-treatment. Therefore, the study presented a serious risk of bias due to deviation from the intended intervention and thus an overall serious risk of bias. The risk of bias assessment for the observational studies is presented in Table 3.

**Table 1:** Summary of findings.

Trial Characteristics (Authors, Year of Publication, Study Design)	Patient Baseline Characteristics (Total Patient Number, Number of Females, Mean Baseline Age, Class II Malocclusion)	Intervention/Comparator Characteristics	Method of Outcome Assessment	Outcomes	Outcomes Results (Herbst/Forsus FRD vs. Cl. II El.: Mean ± SD) (Ns: Non-Significant Mean Difference)
		a. Patients b. Observational period c. Appliance use duration			
Neslon et al. [38] (2000, prospective)	*n* = 36 (F = 0) Herbst: 13.7y, Cl. II el: 13.5y Bilateral Class II malocclusion	a. Herbst (*n* = 18) vs. Cl. II el. (*n* = 18) b. Herbst: 1.1y, Cl. II el.: 1y c. Herbst: 0.5y, Cl. II el.: 1y	Cephalometric radiographs	Short-term dental and skeletal changes	Overjet: −2.1 ± 0.6 mm Overbite: −1.7 ± 0.7 mm Upper Incisors (is-A): −2.8 ± 0.9 mm Lower Incisors (ii-pg): 1.4 ± 0.6 mm Upper molars (A-ms): ns Lower molars (pg-mi): −1.5 ± 0.7 mm Molar correction: ns Maxilla (A-Olp): ns, (NSL/ML°): −1.3 ± 0.5° Mandible (pg-Olp): ns Jaw relationship: (A-pg): 2 ± 0.7 mm, LAFH: −1.1 ± 0.5 mm
Neslon et al. [39] (2007, prospective)	*n* = 30 (F = 0) Herbst: 13.7y, Cl. II el: 13.5y Bilateral Class II malocclusion	a. Herbst (*n* = 15) vs. Cl. II el. (*n* = 15) b. Herbst: 7.1y, Cl. II el.: 9.9y c. Herbst: 0.5y, Cl. II el.: 1y	Cephalometric radiographs	Long-term dental and skeletal changes	Overjet: ns Overbite: ns Upper Incisors (is-A): ns Lower Incisors: (ii-pg): ns, (ILs/NL°): ns, (Ili/ML°): ns Maxilla: (A-Olp): −2.5 ± 1.1 mm, (SNA): ns Mandible: (pg-Olp): ns, (SNB): ns, (NSL/ML): ns Jaw relationship: (A-pg): ns, LAFH:-3.9 ± 1.1 mm
Serbesis-Tsarudis, Pancherz [40] (2008, retrospective)	*n* = 64 (F = 35) Herbst: 12.4y, Cl. II el.: 12.3y Bilateral Class II malocclusion	a. Herbst (*n* = 40) vs. Cl. II el. (*n* = 24) b. Herbst: 2.6y, Cl. II el.: 2.6y c. Herbst: 0.6y, Cl. II el.: 2.6y	Cephalometric radiographs	TMJ and Chin position changes	Mandible: (Pg point): 2.6 ± 1 mm more anteriorly Condylar changes: (Co point): 1.6 ± 0.5 mm more posteriorly
Jones et al. [41] (2008, retrospective)	*n* = 68 (F = 28) Forsus FRD: 12.6y, Cl. II el.: 12.2y Bilateral Class II malocclusion	a. Forsus (*n* = 34) vs. Cl. II el. (*n* = 34) b. Forsus: 2.7y, Cl. II el.: 2.4y c. Forsus: 2.7y, Cl. II el.: 2.4y	Cephalometric radiographs	Dental and skeletal changes	Overjet: ns Upper Incisors: (U1-SN°): ns, (U1-PP): ns Lower Incisors (L1-MP°): ns, (L1-MP): −2.2 ± 1.4 mm Upper molars (U6-PP): ns Lower molars: (L6-MP): ns, (L6): 1.1 ± 0.3 mm (more mesially), (Op-SN°): −0.8 ± 0.8° (clockwise rotation) Molar relationship: (U6/L6): 0.8 ± 0.4 (greater correction) Maxilla (Max): ns Mandible (Mand): ns Jaw relationship (ABCH): ns
Aras, Pasaoglu [46] (2016, RCT)	*n* = 28 (F = 20) Forsus FRD: 14.2 ± 1y, Cl. II el: 13.8 ± 1.2y Class II subdivision	a. Forsus FRD (*n* = 14) vs. Cl. II el. (*n* = 14) b. Forsus FRD: 17.4 ± 0.9 m, Cl. II el: 20.7 ± 1 m c. Forsus FRD: 4.5 ± 0.9 m, Cl. II el: 6.9 ± 1.1 m	Cephalometric radiographs and dental casts measurements	Dental and skeletal changes	Overjet: −0.93 ± 0.4 mm Overbite: ns Upper Incisors: (U1-SN°):2.03 ± 0.7°, (U1-PP): −0.83 ± 0.3 mm Lower Incisors (L1-MP°): ns, (L1-MP): −1.07 ± 1.3 mm Upper molars: (U6-PP°): ns (U6-PP): ns Lower molars: (Op-SN°): −0.88 ± 0.4° (clockwise rotation) Maxilla (SNA°): ns Mandible (SNB°): ns Jaw relationship: (ANB°): ns, (SN-GoGn°): ns Molar correction (dental casts): (Cl. II side): −0.92 ± 0.3 mm Midline correction (dental casts): −0.41 ± 0.2 mm
LeCornu et al. [43] (2013, prospective)	*n* = 14 Herbst: 13y, Cl. II el: 13.4y Bilateral Class II malocclusion	a. Herbst (*n* = 7) vs. Cl. II el. (*n* = 7) b. Herbst: 1.1y, Cl. II el.: 1.5y c. Herbst: 6 to 9 m, Cl. II el.: 1.5y	CBCT	Skeletal jaw changes and Condyle/Glenoid fossa changes	Maxilla: (A-point): −2.42 ± 0.3 mm, (ANS): −1.7 ± 0.5 mm Mandible: (Pg-point): ns, (B-point): 1.14 ± 0.5 mm Condylar changes: anterior surface: (R) 2.52 ± 0.3 mm, (L) 2.94 ± 0.4 mm, posterior surface: (R) 1.74 ± 0.5 mm, (L) 1.35 ± 0.5 mm, Co-point: (R) 1.26 ± 0.3 mm, (L) 1.72 ± 0.3 mm (anterior displacement) Fossa changes: anterior fossa: (R) 3.19 ± 0.3 mm, (L) 2.74 ± 0.4 mm, (anterior displacement/bone resorption) posterior fossa: (R) ns, (L) 2.2 ± 0.6 mm (anterior displacement/bone apposition)
Atresh et al. [44] (2018, prospective)	*n* = 27 (F = 17) Herbst brachyfacial: 13.3y, Herbst mesofacial 12.7y, Cl. II el.: 13.7y Bilateral Class II malocclusion	a. Herbst brachyfacial (*n* = 8), Herbst mesofacial (*n* = 8), Cl. II el. (*n* = 11) b. Herbst brachyfacial: 27.6 m, Herbst mesofacial 27.1 m, Cl. II el.: 22.8 m c. Herbst brachyfacial: 7.2 m, Herbst Mesofacial: 7.9 m, Cl. II el.: 22.8 m	CBCT	Skeletal jaw changes and Condyle/Glenoid fossa changes	Maxilla: Brachyfacial Herbst (ANS): −1.4 ± 0.6 mm (inferior displacement), Mesofacial Herbst (ANS): −1.9 ± 0.5 mm (inferior displacement) Mandible: (Pg-point): ns, Brachyfacial Herbst (B-point): −2.5 ± 1.2 mm (inferior displacement), Mesofacial Herbst (B-point): −2.7 ± 1 mm (inferior displacement) Condylar changes: ○ 3D condylar geometric center position: ns ○ (R) Condylion AP&SI & (L) Condylion AP: ns ○ Brachyfacial Herbst (L) Condylion SI: −0.81 ± 0.4 mm (inferior displacement), ○ Mesofacial Herbst (L) Condylion SI: −0.77 ± 0.3 mm (inferior displacement), ○ Mesofacial Herbst Gonial angle (Co-Go-Me°): (R) 1.59 ± 0.6° Fossa changes anterior fossa: ○ Brachyfacial Herbst: (R) AP: −1.25 ± 0.5 mm (posterior displacement), (R) SI, (L) AP&SI: ns ○ Mesofacial Herbst: (R) AP:ns, (R) SI: −0.64 ± 0.2 mm (inferior displacement), (L) AP&SI: ns posterior fossa: ○ Brachyfacial Herbst: (L) SI: 0.81 ± 0.4 mm, (R) AP&SI & (L) AP: ns ○ Mesofacial Herbst: ns
Wei et al. [42] (2020, retrospective)	*n* = 31 (F = 21) Herbst: 13y, Cl. II el: 13.5y, Bilateral Class II malocclusion	a. Herbst (*n* = 20), Cl. II el. (*n* = 11) b. Herbst: 2.5y, Cl. II el: 1.9y c. Herbst: 7.8 m Cl. II el: 22.9 m	CBCT	Condylar changes	Condylar changes during total treatment (Herbst vs. Cl. II elastics: ○ condyle superior point (vertical growth): 2.27 ± 1 mm, ○ condyle posterior point (posterior growth): 0.96 ± 0.5 mm Condylar changes during phase 1 and 2 of Herbst appliance treatment: ○ condyle superior point (vertical growth): (orthopedic phase: 2.97 ± 1.87 mm, subsequent orthodontic phase: 2.99 ± 1.89 mm) ○ condyle posterior point (posterior growth): (orthopedic phase: 1.52 ± 0.99 mm, subsequent orthodontic phase: 0.45 ± 0.98 mm)
Nindra et al. [45] (2021, retrospective)	*n* = 30 (F = 16) Herbst (splint): 13y 2 m, Cl. II el: 14y 5 m Bilateral Class II malocclusion	a. Herbst (*n* = 15), Cl. II el. (*n* = 15) b. Herbst: 8–10 m Cl. II el.: 14–16 m c. Herbst: 8–10 m Cl. II el.: 14–16 m	CBCT	Condyle and Glenoid fossa changes	Condylar changes: condyle volume: (R) 111 ± 27.1 mm^3^, (L) 127.8 ± 22.8 mm^3^, condyle height: (R) 1.35 ± 0.3 mm, (L)1.21 ± 0.3 mm, condylar position in the fossa: ns Glenoid fossa changes: ns TMJ space: ns

**Table 2:** Risk of bias assessment for Randomized clinical trials.

Study	Bias Arising from the Randomization Process	Bias Due to Deviations from Intended Interventions	Bias Due to Missing Outcome Data	Bias in the Measurement of the Outcome	Bias in the Selection of the Reported Result	Overall Risk of Bias
Aras, Pasaoglu [46] (2016)	Low for all outcomes (matched randomization was used)	Low for all outcomes (no bias due to departure from the intended intervention is expected)	Low for all outcomes (data were reasonably complete)	Low for all outcomes (objective method of outcome assessment, outcome assessor was blinded)	Low for all outcomes (all reported results correspond to intended outcome)	Low for all outcomes

**Table 3:** Risk of bias assessment for non-randomized studies of interventions.

Study	Bias Due to Confounding	Bias in Selection of Participants into the Study	Bias in Classification of Interventions	Bias Due to Deviations from Intended Interventions	Bias Due to Missing Data	Bias in the Measurement of The Outcomes	Bias in the Selection of the Reported Result	Overall Risk of Bias
Neslon et al. [38] (2000)	Low for all outcomes (Patients were matched based on malocclusion, age, and somatic maturity)	Low for all outcomes (All eligible patients were selected and start of intervention and follow-up coincide)	Low for all outcomes (well defined intervention status)	Low for all outcomes (no bias due to departure from the intended intervention is expected)	Low for all outcomes (no missing outcome data)	Low for all outcomes (all pre-specified variables were measured)	Low for all outcomes (all reported results correspond to intended outcome)	Low for all outcomes
Neslon et al. [39] (2007)	Low for all outcomes (Patients were matched based on malocclusion, age, and somatic maturity)	Low for all outcomes (All eligible patients were selected and start of intervention and follow-up coincide)	Low for all outcomes (well defined intervention status)	Low for all outcomes (no bias due to departure from the intended intervention is expected)	Low for all outcomes (no missing outcome data)	Low for all outcomes (all pre-specified variables were measured)	Low for all outcomes (all reported results correspond to intended outcome)	Low for all outcomes
Serbesis-Tsarudis, Pancherz [40] (2008)	Low for all outcomes (patients had similar malloclusion, age, treatment time, and eligibility criteria)	Low for all outcomes (All eligible patients were selected and start of intervention and follow-up coincide)	Low for all outcomes (well defined intervention status)	Low for all outcomes (no bias due to departure from the intended intervention is expected)	Low for all outcomes (no missing outcome data)	Low for all outcomes (objective method of outcome assessment, any error is unrelated to intervention status)	Low for all outcomes (all reported results correspond to intended outcome)	Low for all outcomes
Jones et al. [41] (2008)	Low for all outcomes (Patients were matched based on malocclusion, gender, age, treatment duration, and angles: ANB, L1-GoMe, SN-GoMe)	Low for all outcomes (All eligible patients were selected and start of intervention and follow-up coincide)	Low for all outcomes (well defined intervention status)	Serious for all outcomes (Forsus FRD patients could have used Cl. II elastics prior to Forsus, or for retention after Forsus use)	Low for all outcomes (no missing outcome data)	Low for all outcomes (objective method of outcome assessment, any error is unrelated to intervention status)	Low for all outcomes (all reported results correspond to intended outcome)	Serious for all outcomes
LeCornu et al. [43] (2013)	Low for all outcomes (Patients were matched based on cervical vertebral maturity, jaw relationship and malloclusion)	Low for all outcomes (All eligible patients were selected and start of intervention and follow-up coincide)	Low for all outcomes (well defined intervention status)	Low for all outcomes (no bias due to departure from the intended intervention is expected)	Low for all outcomes (no missing outcome data)	Low for all outcomes (all pre-specified variables were measured)	Low for all outcomes (all reported results correspond to intended outcome)	Low for all outcomes
Atresh et al. [44] (2018)	Low for all outcomes (Patients were matched based on similar baseline characteristics)	Low for all outcomes (All eligible patients were selected and start of intervention and follow-up coincide)	Low for all outcomes (well defined intervention status)	Low for all outcomes (no bias due to departure from the intended intervention is expected)	Low for all outcomes (data were reasonably complete)	Low for all outcomes (objective method of outcome assessment, any error is unrelated to intervention status)	Low for all outcomes (all reported results correspond to intended outcome)	Low for all outcomes
Wei et al. [42] (2020)	Low for all outcomes (Patients were matched based on similar baseline characteristics)	Low for all outcomes (All eligible patients were selected and start of intervention and follow-up coincide)	Low for all outcomes (well defined intervention status)	Low for all outcomes (no bias due to departure from the intended intervention is expected)	Low for all outcomes (data were reasonably complete)	Low for all outcomes (objective method of outcome assessment, any error is unrelated to intervention status)	Low for all outcomes (all reported results correspond to intended outcome)	Low for all outcomes
Nindra et al. [45] (2021)	Low for all outcomes (Patients were matched based on similar baseline characteristics)	Low for all outcomes (All eligible patients were selected and start of intervention and follow-up coincide)	Low for all outcomes (well defined intervention status)	Low for all outcomes (no bias due to departure from the intended intervention is expected)	Low for all outcomes (data were reasonably complete)	Low for all outcomes (objective method of outcome assessment, any error is unrelated to intervention status)	Low for all outcomes (all reported results correspond to intended outcome)	Low for all outcomes

### 3.4. Results of Individual Studies

The results of the included studies (briefly presented in Table 1) are analyzed below:

### 3.5. Overjet

The additional short-term average overjet decrease of 2 mm (*p*-value < 0.01) with Herbst relative to the Class II elastics [38] was diminished in the long term [39] (*p*-value > 0.05). Moreover, an additional average overjet decrease of 0.93 mm (*p*-value = 0.02) was observed in one study [46] comparing Forsus to Class II elastics, which was not different in another [41] (*p*-value > 0.05).

### 3.6. Overbite

Similarly, the additional short-term average decrease of 1.7 mm (*p*-value < 0.05) with Herbst relative to Class II elastics [38] was diminished in the long term [39] (*p*-value > 0.05). No difference in overbite changes was also found between Forsus and Class II elastics [46] (*p*-value > 0.05).

### 3.7. Upper Incisor Inclination and Position

In the short-term, the upper incisors were found 2.8 mm less posteriorly (is-A, *p*-value < 0.01) with Herbst relative to Class II elastics [38], but there was no long-term difference between them [39] (is-A, ILs/NL, *p*-value > 0.05). There was also 2 mm and 0.83 mm less retroclination relative to the cranial base (U1-SN, *p*-value = 0.06) and palatal plane (U1-PP, *p*-value = 0.014), respectively, between Forsus and Class II elastics [46].

### 3.8. Lower Incisor Inclination

Lower incisors were found 1.4 mm less proclined (ii-pg, *p*-value < 0.05) with Herbst relative to Class II elastics in the short-term [38], but not in the long-term [39] (ii-pg, Ili/ML, *p* > 0.05).

### 3.9. Molar Relationship

There was no difference in molar correction (ms-mi, *p*-value > 0.05) and upper molar movement (A-ms, *p*-value > 0.05) between Herbst and Class II elastics after one year of treatment [38]. Conversely, the lower molars moved 1.5 mm further mesially with the Herbst (pg-mi, *p*-value > 0.05) mainly due to the greater skeletal improvement in jaw relationship [38].

There was 0.8 mm greater mean molar correction (U6/L6, *p*-value = 0.03) and 1.1 mm more mesial lower molar movement (L6, *p*-value < 0.001) with Forsus compared to Class II elastics, without any changes in the upper molar position [41]. Similarly, a 0.92 mm greater mean molar correction in the Class II side (molar correction, *p*-value = 0.01) of Class II subdivision patients and an unchanged upper molar position was also observed with Forsus in another study [46]. Finally, there was a 0.8° to 0.88° greater average clockwise occlusal plane rotation (Op/SN°, Occ/SN°, *p*-value < 0.05) in patients treated with Forsus [41,46].

### 3.10. Maxillary Changes

Point A was found retruded by 2.42 mm (point A, *p*-value < 0.05) and 2.5 mm (A-Olp, *p*-value < 0.05) with Herbst compared Class II elastics in two studies [39,43], but no differences were observed in two other studies [38,44] (*p*-value > 0.05). Additionally, there was a 1.7 mm greater mean posterior displacement of the ANS in one study [43] (*p*-value < 0.05), and a 1.4 mm greater mean superior displacement (*p*-value = 0.01) in brachyfacial patients and 1.9 mm in mesofacial patients (*p*-value = 0.001) with Herbst compared to Class II elastics in another study [44]. Furthermore, there was no SNA angle° difference (*p*-value > 0.05) between Herbst or Forsus and Class II elastics [41,46].

### 3.11. Mandibular Changes

There was a 2.6 mm greater mean anterior pogonion displacement (Pg point, *p*-value < 0.01) with Herbst compared to Class II elastics in one study [40], but not any anteroposterior [38,39,43,44] or vertical [38,39,43,44,47] differences (*p*-value > 0.05) in all the other studies. Moreover, B point was found 1.14 mm more anteriorly (*p*-value < 0.05) in one study [43], and 2.5 mm and 2.7 mm more inferiorly in brachyfacial (*p*-value = 0.03) and mesofacial patients (*p*-value = 0.02), respectively in another study [44] with Herbst compared to Class II elastics. Finally, there were no SNB angle° differences (*p*-value > 0.05) between Herbst or Forsus and Class II elastics [41,46].

### 3.12. Condylar Changes

When the X-rays were superimposed on the mandible, an arbitrary condylar point was found 1.6 mm more posteriorly (Co point, *p*-value < 0.001) with Herbst compared to Class II elastics in a study [40], while in another study [42] a posterior condylar point was 0.96 mm more posteriorly and a superior condylar point 2.27 mm more superiorly.

When cranial base 3D superimpositions were used, patients treated with Herbst, compared to Class II elastics, had a greater anterior displacement of both condyles in one study [43] (Right Co: 1.26 ± 0.3 mm, *p*-value < 0.05, Left Co: 1.72 ± 0.3 mm, *p*-value < 0.05), a greater inferior displacement of the left condyle and no other sagittal/vertical differences in another study [44](brachyfacial/left condylion: 0.81 ± 0.4 mm, *p*-value = 0.03, mesofacial/left condylion: 0.77 ± 0.3 mm, *p*-value = 0.04), and a greater volume (Right condyle: 111 ± 27.1 mm^3^, *p*-value = 0.002, Left condyle: 127.8 ± 22.8 mm^3^, *p*-value = 0.001) and height increase (Right condyle: 1.35 ± 0.3 mm, *p*-value = 0.001, Left condyle: 1.21 ± 0.3 mm, *p*-value = 0.004) of both condyles in a third study [45].

### 3.13. Glenoid Fossae Changes

Patients treated with Herbst, compared to Class II elastics, had a greater anterior displacement of both anterior (Right: 3.19 ± 0.3 mm, *p*-value < 0.05, Left: 2.74 ± 0.4 mm, *p*-value < 0.05) and left posterior glenoid fossae (2.2 ± 0.6 mm, *p*-value < 0.05) in one study [43], and a greater posterior (brachyfacial: −1.25 ± 0.5 mm, *p*-value = 0.01) or inferior (mesofacial: −0.64 ± 0.2 mm, *p*-value = 0.03) displacement of the right anterior glenoid fossa along with a greater mean inferior displacement the left posterior fossa (brachyfacial: 0.81 ± 0.4 mm, *p*-value = 0.02) in a second study [44]. Finally, no intergroup glenoid fossae or condyle position differences were observed in a third study [42].

### 3.14. Jaw Relationship

There was only a short-term 2 mm greater sagittal improvement in jaw base (*p*-value < 0.01) with Herbst compared to Class II elastics [38]. Additionally, there was a 1.1 mm short-term (*p*-value < 0.05) and 3.9 mm long-term (*p*-value < 0.001) [39] greater decrease in lower anterior facial height with the Herbst relative to Class II elastics, while no differences in maxillary inclination to the mandibular plane were observed [38]. Finally, no differences were found between Forsus and Class II elastics [41,46].

### 3.15. Results of Meta-Analysis

Three studies [39,41,46] were included in the meta-analysis of overjet, overbite, upper and lower incisor inclination, two studies [39,46] in SNA and SNB angles °, and two studies [43,44] in the anteroposterior changes of A point, ANS, B point, Pg point, right and left Co points, right and left anterior glenoid fossa, and right and left gonial angle. The most recent study by Nelson et al. [39] was included in the meta-analyses due to the larger follow-up period [38]. Additionally, the brachyfacial and mesofacial groups of the study by Atresh et al. [44] were incorporated separately in the meta-analysis of the relative outcomes but the Class II elastics group (*n* = 11) was separated into five and six patients to prevent double-counting [31].

#### 3.15.1. Overjet

**Figure 2:**
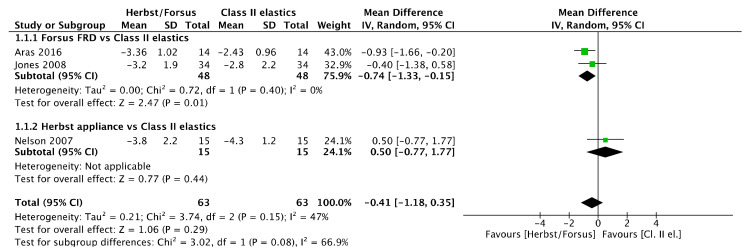
Forest plot of the overjet changes using the random effects model [39,41,46].

No overjet difference (Figure 2) was observed between Herbst/Forsus and Class II elastics [mean difference (95% CI): −0.41 mm (−1.18, 0.35), I^2^ = 47%] and between Herbst and Class II elastics [mean difference (95% CI): 0.5 mm (−0.77, 1.77)]. However, there was a difference favoring Forsus compared to Class II elastics [mean difference (95% CI): −0.74 mm (−1.33, −0.15), I^2^ = 0%].

#### 3.15.2. Overbite

**Figure 3:**
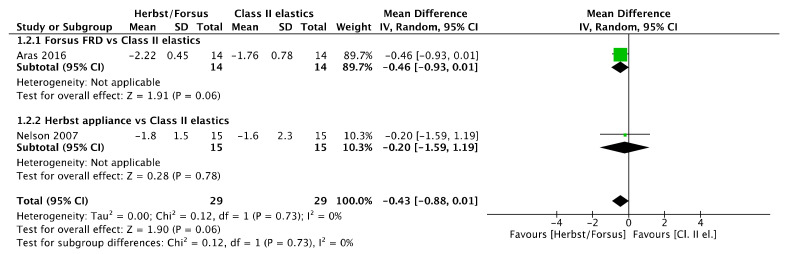
Forest plot of the overbite changes using the random effects model [39,46].

No overbite difference (Figure 3) was observed in overbite between Herbst/Forsus and Class II elastics [mean difference (95% CI): −0.43 mm (−0.88, 0.01), I^2^ = 0%], between Herbst and Class II elastics [mean difference (95% CI): −0.2 mm (−1.59, 1.19)], and between Forsus and Class II elastics [mean difference (95% CI): −0.46 mm (−0.93, 0.01)].

#### 3.15.3. Upper Incisor Inclination

**Figure 4:**
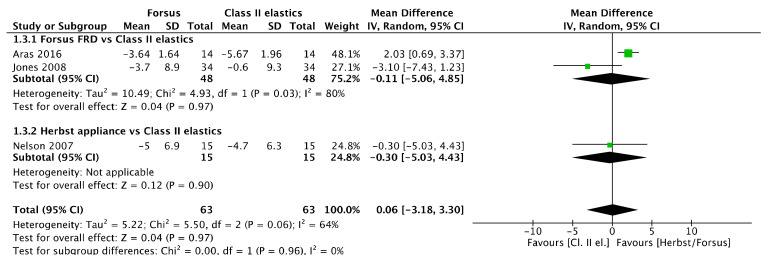
Forest plot of the upper incisor inclination changes using the random effects model [39,41,46].

No upper incisor inclination difference (Figure 4) was observed between Herbst/Forsus and Class II elastics [mean difference (95% CI): 0.06° (−3.18, 3.30), I^2^ = 64%], between Herbst appliance and Class II elastics [mean difference (95% CI): −0.30° (−5.03, 4.43)], and between Forsus and Class II elastics [mean difference (95% CI): −0.11° (−5.06, 4.85), I^2^ = 80%].

#### 3.15.4. Lower Incisor Inclination

**Figure 5:**
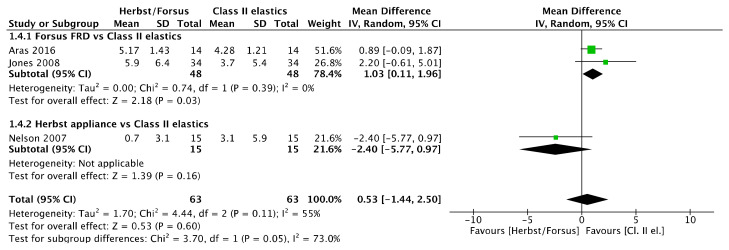
Forest plot of the lower incisor inclination changes using the random effects model [39,41,46].

No lower incisor inclination difference (Figure 5) was observed between Herbst/Forsus and Class II elastics [mean difference (95% CI): 0.53° (−1.44, 2.50), I^2^ = 55%] and between Herbst and Class II elastics [mean difference (95% CI): −2.40° (−5.77, 0.97)]. However, there was a greater lower incisor inclination with Forsus than with Class II elastics [mean difference (95% CI): 1.03° (0.11, 1.96), I^2^ = 0%].

#### 3.15.5. Maxillary Changes

**Figure 6:**
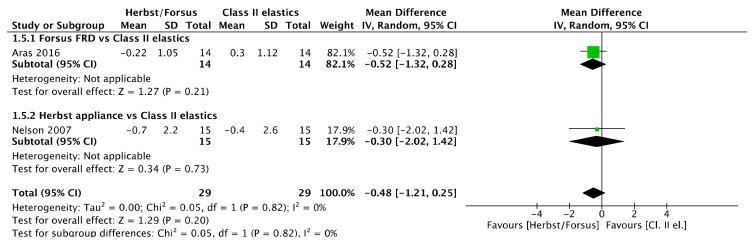
Forest plot of the SNA angle changes using the random effects model [39,46].

No SNA angle (Figure 6) difference was observed between Herbst/Forsus and Class II elastics [mean difference (95% CI): −0.48° (−1.21, 0.25), I^2^ = 0%], between Herbst and Class II elastics [mean difference (95% CI): −0.30° (−2.02, 1.42)], and between Forsus and Class II elastics [mean difference (95% CI): −0.52° (−1.32, 0.28)].

**Figure 7:**
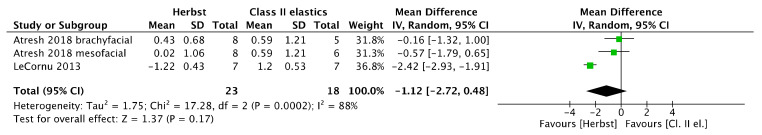
Forest plot of the A point anteroposterior changes using the random effects model [43,44].

**Figure 8:**
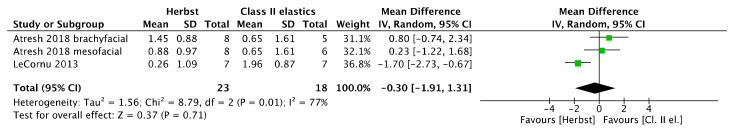
Forest plot of the ANS point anteroposterior changes using the random effects model [43,44].

Finally, no anteroposterior differences were found between Herbst and Class II elastics group in point A (Figure 7) [mean difference (95% CI): −1.12 mm (−2.72, 0.48), I^2^ = 88%] and ANS (Figure 8) [mean difference (95% CI): −0.3 mm (−1.91, 1.31), I^2^ = 77%].

#### 3.15.6. Mandibular Changes

**Figure 9:**
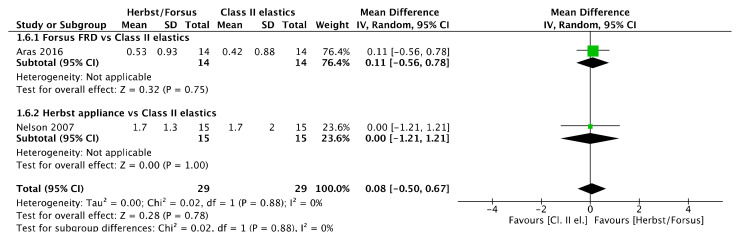
Forest plot of the SNB angle changes using the random effects model [39,46].

No SNB angle (Figure 9) difference was observed between Herbst/Forsus and Class II elastics [mean difference (95% CI): 0.08° (−0.50, 0.67), I^2^ = 0%], between Herbst and Class II elastics [mean difference (95% CI): 0° (−1.21, 1.21)], and between Forsus and Class II elastics [mean difference (95% CI): 0.11° (−0.56, 0.78)].

**Figure 10:**
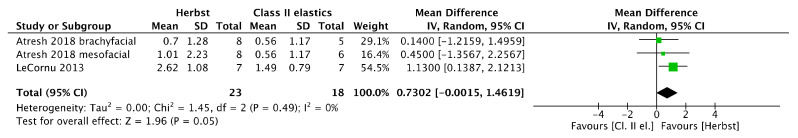
Forest plot of the B point anteroposterior changes using the random effects model [43,44].

**Figure 11:**
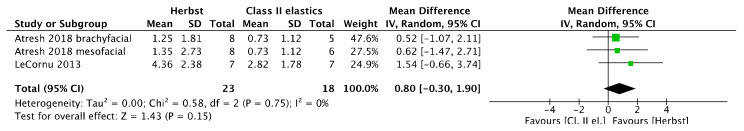
Forest plot of Pg point anteroposterior changes using the random effects model [43,44].

**Figure 12:**
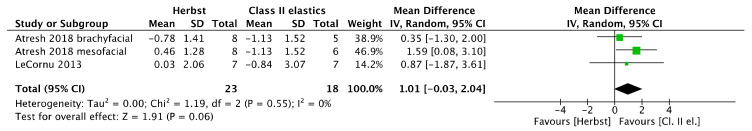
Forest plot of the right gonial angle changes using the random effects model [43,44].

**Figure 13:**
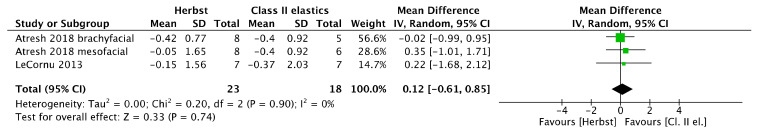
Forest plot of the left gonial angle changes using the random effects model [43,44].

**Figure 14:**
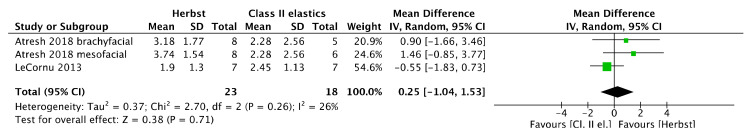
Forest plot of the right mandibular corpus length changes using the random effects model [43,44].

**Figure 15:**
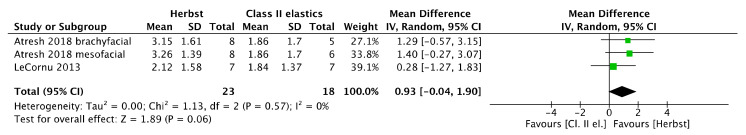
Forest plot of the left mandibular corpus length changes using the random effects model [43,44].

**Figure 16:**
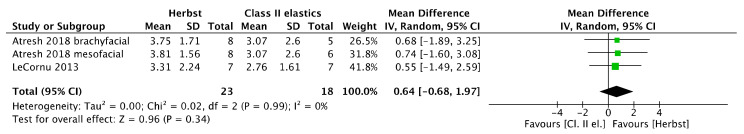
Forest plot of the left mandibular ramus height changes using the random effects model [43,44].

No anteroposterior differences were also observed between Herbst and Class II elastics regarding B point (Figure 10) [mean difference (95% CI): 0.73 mm (−0.002, 1.46), I^2^ = 0%], Pg point (Figure 11) [mean difference (95% CI): 0.8 mm (−0.3, 1.9), I^2^ = 0%], right gonial angle (Figure 12) [mean difference (95% CI): 1.01° (−0.03, 2.04), I^2^ = 0%], left gonial angle (Figure 13) [mean difference (95% CI): 0.12° (−0.61, 0.85), I^2^ = 0%], right mandibular corpus length (Figure 14) [mean difference (95% CI): 0.25 mm (−1.04, 1.53), I^2^ = 26%], left mandibular corpus length (Figure 15) [mean difference (95% CI): 0.93 mm (−0.04, 1.90), I^2^ = 0%], and left mandibular ramus height (Figure 16) [mean difference (95% CI): 0.64 mm (−0.68, 1.97), I^2^ = 0%].

**Figure 17:**
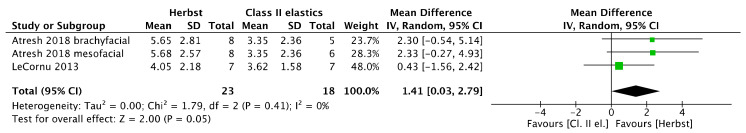
Forest plot of the right mandibular length changes using the random effects model [43,44].

**Figure 18:**
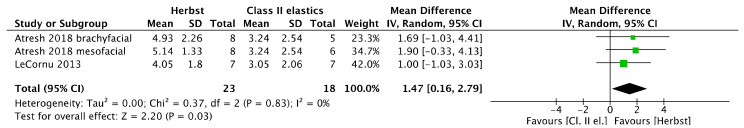
Forest plot of the left mandibular length changes using the random effects model [43,44].

**Figure 19:**
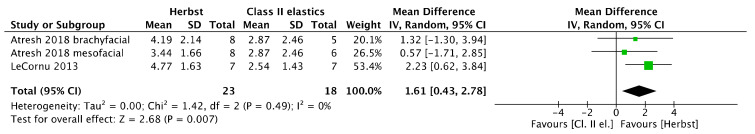
Forest plot of the right mandibular ramus height changes using the random effects model [43,44].

Significant differences favoring Herbst compared to Class II elastics were observed for right mandibular length (Co-Gn) (Figure 17) [mean difference (95% CI): 1.41 mm (0.03, 2.79), I^2^ = 0%], left mandibular length (Co-Gn) (Figure 18) [mean difference (95% CI): 1.47 mm (0.16, 2.79), I^2^ = 0%], and right mandibular ramus height (Co-Go’) (Figure 19) [mean difference (95% CI): 1.61 mm(0.43, 2.78), I^2^ = 0%].

#### 3.15.7. Condylar and Glenoid Fossa Changes

**Figure 20:**
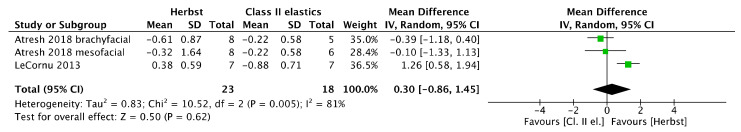
Forest plot of the right Co point anteroposterior changes using the random effects model [43,44].

**Figure 21:**
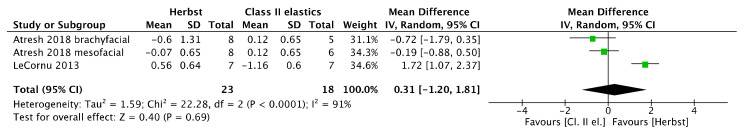
Forest plot of the left Co point anteroposterior changes using the random effects model [43,44].

**Figure 22:**
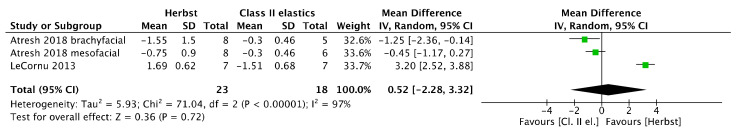
Forest plot of right anterior glenoid fossa anteroposterior changes using the random effects model [43,44].

**Figure 23:**
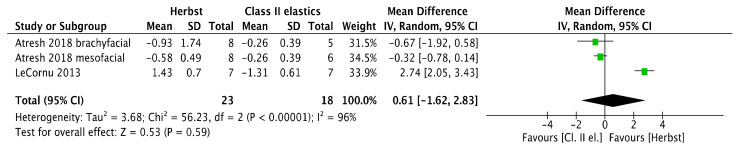
Forest plot of the left anterior glenoid fossa anteroposterior changes using the random effects model [43,44].

**Figure 24:**
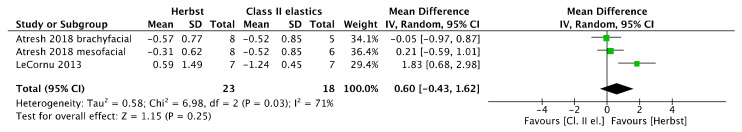
Forest plot of right posterior glenoid fossa anteroposterior changes using the random effects model [43,44].

**Figure 25:**
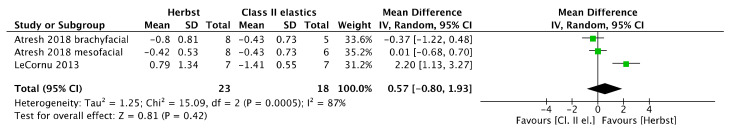
Forest plot of left posterior glenoid fossa anteroposterior changes using the random effects model [43,44].

No anteroposterior differences were observed between Herbst and Class II elastics regarding right Co point (Figure 20) [mean difference (95% CI): 0.3 mm (−0.86, 1.45), I^2^ = 81%], left Co point (Figure 21) [mean difference (95% CI): 0.31 mm (−1.20, 1.81), I^2^ = 91%], right anterior glenoid fossa (Figure 22) [mean difference (95% CI): 0.52 mm (−2.28, 3.32), I^2^ = 97%], left anterior glenoid fossa (Figure 23) [mean difference (95% CI): 0.61 mm (−1.62, 2.83), I^2^ = 96%], right posterior glenoid fossa (Figure 24) [mean difference (95% CI): 0.6 mm (−0.43, 1.62), I^2^ = 71%]), and left posterior glenoid fossa (Figure 25) [mean difference (95% CI): 0.57 mm(−0.8, 1.93), I^2^ = 87%].

### 3.16. Sensitivity Analysis

**Figure 26:**
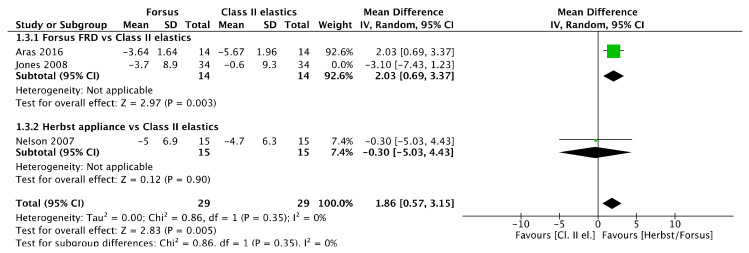
Forest plot of the sensitivity analysis on upper incisor inclination changes using the random effects model [39,46].

A sensitivity analysis was performed for upper and lower incisor inclination by removing the study with the serious overall risk of bias [41]. Accordingly, there was a 1.86° greater upper incisor retroclination (Figure 26) with Class II elastics compared to Herbst/Forsus [mean difference (95% CI): 1.86° (0.57, 3.15), I^2^ = 0%], 2.03° greater compared to Forsus [mean difference (95% CI): 2.03° (0.69, 3.37)], while there was no difference with Herbst [mean difference (95% CI): −0.30° (−5.03, 4.43)].

**Figure 27:**
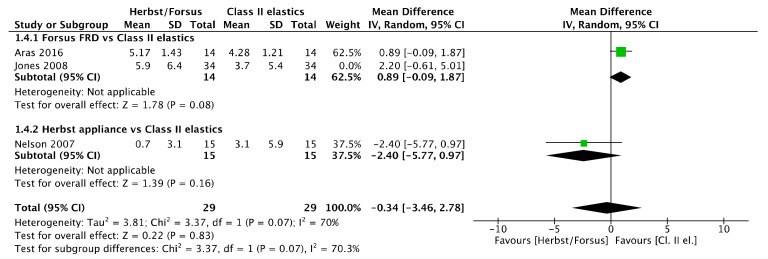
Forest plot of the sensitivity analysis on lower incisor inclination changes using the random effects model [39,46].

However, the study [41] was not removed from the lower incisor inclination meta-analysis (Figure 27) because heterogeneity was increased, while the results remained not significantly different [mean difference (95% CI): −0.34° (−3.46, 2.78), I^2^ = 70%).

### 3.17. Reporting Biases Assessment

An evaluation for the existence of reporting bias (including publication bias) was not possible, due to the limited number of included studies (two or three studies) for the meta-analysis of each outcome.

### 3.18. Certainty Assessment

The quality of evidence quality according to GRADE [32] was judged to be high for overbite, SNA angle, SNB angle, and upper incisor inclination while it was judged low and very low for all the other outcomes. The GRADE summary of findings is presented in Table 4.

## 4. Discussion

### 4.1. Summary of Evidence

Our study aimed to summarize the evidence on the dentoskeletal effects of Herbst and Forsus, compared to Class II elastics for treating Class II malocclusion growing patients. Eight observational studies [38,39,41,42,43,44,45,47] and one randomized-controlled trial [46] were included, while seven compared Herbst to Class II elastics and two compared Forsus to Class II elastics. The risk of bias was low for most studies [38,39,42,43,44,45,47]. Jones et al. [41] study was at serious overall risk of bias because the patients treated with Forsus could have used Class II elastics before Forsus assignment and thus a bias due to the deviations from the intended interventions could arise.

According to our meta-analysis, there was a high level of evidence showing less post-treatment upper incisor retroclination and no differences in overbite, SNA, and SNB angle, with Herbst/Forsus compared to Class II elastics. A low level of evidence also suggested no differences in lower incisor inclination and overjet correction. In the subgroup analysis, Forsus was found more beneficial in overjet correction and in controlling upper incisor inclination.

Furthermore, a low and very low level of evidence suggested no anteroposterior differences between Herbst and Class II elastics in maxillary, condylar, glenoid fossae, and most mandibular changes, except for a greater increase in mandibular length (approximately 1.5 mm bilaterally) and right mandibular ramus height (1.6 mm) with Herbst, based on a low quality of evidence.

### 4.2. Strengths and Limitations

PRISMA guidelines [33] were strictly followed throughout this study and a meta-analysis was performed for every prespecified outcome. Additionally, it is the first systematic review and meta-analysis to include 3D-superimpositions data for Herbst and Class II elastics comparison. Conversely, only one randomized clinical trial [46] and mostly observational studies [38,39,42,43,44,45] were included, and therefore the evidence for most outcomes was low and very low. Moreover, most studies used different cephalometric or three-dimensional analyses, and thus only two or three studies could be used for each synthesis. Finally, the limited number of included studies, their small samples, and the wide range of patient growth potential relative to the pubertal growth peak [47,48] could explain the high heterogeneity of many outcomes.

### 4.3. Critical Appraisal of the Results in the Context of other Evidence

There seem to be more similarities than differences between Herbst or Forsus and Class II elastics, indicating that regardless of the method and the magnitude of applying backward forces on the maxillary dentition and forward forces on the mandibular dentition the outcomes will be equivalent. Nevertheless, greater upper incisor control (<2 retroclination) was found with the functional appliances compared to Class II elastics, suggesting that despite the minimal difference, a slightly less backward force was applied on the upper teeth. On the other hand, the lighter continuous forces of the Class II elastics were found to be equally effective in all the other dental (overjet, overbite, lower incisor inclination) and skeletal parameters (SNA, SNB) with those created by the mandibular advancement caused by these functional appliances.

Interestingly, despite that the meta-analysis of the studies with 2D cephalometric radiographs showed no difference in mandibular growth (SNB angle) between Herbst and Class II elastics, the meta-analysis of the studies with that performed 3D linear measurements showed that Herbst caused an additional bilateral mandibular growth (Co, Gn) of approximately 1.5 mm. This can be explained by the limitations of 2D cephalometric X-rays (difficulty in the identification of landmarks due to overlapping structures, magnification, distortion, and patient positioning) compared to the more accurate 3D X-rays which give the operator the ability to accurately identify the landmarks on the mandible and perform 3D superimpositions [43,49,50]. Moreover, despite not finding any difference in the anteroposterior position of the condyle and glenoid fossa with Herbst, additional condylar volume and height were observed with Herbst in one study [45] indicating that vertical condylar changes could have also taken place. This vertical condylar growth could have happened from the remodeling of the condyle due to its disarticulation in the fossa caused by the Herbst mandibular advancement. This hypothesis also agrees with our findings of the additional bilateral mandibular length growth (Co-Gn), the additional right mandibular ramus height increase (Co-Go’), and the non-significantly different mandibular corpus length (Go-Gn) found with Herbst compared to Class II elastics in our study.

Regarding the Class II molar correction, Herbst and Class II elastics were equally able to correct the Class II molar relationship, but Herbst could move the lower molars more mesially (1.5 mm) [38,39]. Similarly, Forsus achieved a greater overjet reduction and was more effective than Class II elastics in mean molar correction (≃1 mm) due to the more mesial lower molar movement (1 mm) [41,46]. These findings could be explained by the disocclusion of the dentition and the greater force application on the lower teeth caused by Herbst or Forsus compared to Class II elastics.

### 4.4. Clinical Implications and Future Research

Equivalent dentoskeletal effects are to be expected from Herbst, Forsus, and Class II elastics on the Class II malocclusion correction, except for a slightly greater upper incisor control with functional appliances.

Forsus had equivalent dentoskeletal effects with Class II elastics but achieved a slightly greater (≃1 mm) molar correction, upper incisor control, and overjet reduction [41,46]. Thus, Forsus might be more effective in correcting a more severe Class II molar relationship.

Moreover, Herbst, compared to Class II elastics, could equally correct the Class II malocclusion [38,39], but despite the 1.5 mm additional short-term mandibular length growth found in our study and the condylar growth [42,45], and fossae remodeling [43,44,45] found in other studies, no sagittal long-term skeletal differences [39] (SNA, SNB angles) are to be expected. Therefore, the clinician should decide if the potentially additional vertical short-term growth acceleration with the Herbst could be more beneficial depending on the severity of the class II malocclusion of each patient.

Despite finding a greater 1.5 mm bilateral increase of mandibular length with Herbst than with elastics, no difference in Pogonion projection was found, maybe because of the counterclockwise mandibular rotation due to vertical growth or dentition changes [42]. Therefore, a study comparing vertical changes between Herbst and class II elastics using three-dimensional superimposition could be useful. Furthermore, high-quality randomized clinical trials with larger samples comparing these appliances are required to provide definitive answers regarding their effectiveness.

## 5. Conclusions

- Comparing Herbst appliance with Class II elastics, Herbst is equally effective as class II elastics in molar correction but causes a more mesial lower molar movement. Apart from an additional bilateral increase in mandibular length (1.5 mm), no other dental and anteroposterior skeletal differences exist.
- A clinical decision regarding the potential for growth acceleration using the Herbst appliance compared to Class II elastics should be made depending on the severity of the Class II malocclusion.
- Comparing Forsus appliance to Class II elastics, Forsus has equivalent dental effects to Class II elastics, but it is more effective in molar correction, overjet reduction, and controlling of the upper incisor inclination.
- High-quality randomized clinical trials with larger sample sizes should be performed to have definitive information about the effectiveness of these treatment methods.

## Figures and Tables

**Table 4:** GRADE summary of findings for all the outcomes.

Outcomes	Anticipated Mean Post-Treatment Difference Herbst/Forsus FRD vs. Class II Elastics (95% CI)	No. of Participants (Studies)	Certainty of Evidence (GRADE)
Overjet	The mean difference in overjet was 0.41 mm less in the intervention group (1.18, 0.35)	126 participants (1 RCT, 2 observational studies)	⊕⊕OO Low [39,41,46] **
Overbite	The mean difference in overbite was 0.43 mm less in the intervention group (−0.88, 0.01)	58 participants (1 RCT, 1 observational study)	⊕⊕⊕⊕ High [39,46], **
Upper incisor inclination	The mean difference in upper incisor inclination was 1.86° less in the intervention group (0.57, 3.15)	58 participants (1 RCT, 1 observational study)	⊕⊕⊕⊕ High [39,46] **
Lower incisor inclination	The mean difference in upper incisor inclination was 0.53° more in the intervention group (−1.44, 2.50)	126 participants (1 RCT, 2 observational studies)	⊕⊕OO Low [43,44], **
SNA angle	The mean difference in SNA angle was 0.48° less in the intervention group (1.21, 0.25)	58 participants (1 RCT, 1 observational study)	⊕⊕⊕⊕ High [39,46],**
A point (AP change)	The mean difference in A point was 1.12 mm less anteriorly in the intervention group (−2.72, 0.48)	41 participants (2 observational studies)	⊕OOO Very Low [43,44], *
ANS point (AP change)	The mean difference in ANS point was 0.3 mm less anteriorly in the intervention group (−1.91, 1.31)	41 participants (2 observational studies)	⊕OOO Very Low [43,44], *
SNB angle	The mean difference in SNB angle was 0.08° more in the intervention group (−0.50, 0.67)	58 participants (1 RCT, 1 observational study)	⊕⊕⊕⊕ High [39,46], **
B point (AP change)	The mean difference in B point was 0.73 mm more anteriorly in the intervention group (−0.002, 1,46)	41 participants (2 observational studies)	⊕⊕OO Low [43,44], *
Pg point (AP change)	The mean difference in Pg point was 0.8 mm more anteriorly in the intervention group (−0.3, 1.9)	41 participants (2 observational studies)	⊕⊕OO Low [43,44], *
right gonial angle	The mean difference in right gonial angle was 1.01° greater in the intervention group (−0.03, 2.04)	41 participants (2 observational studies)	⊕⊕OO Low [43,44], *
left gonial angle	The mean difference in left gonial angle was 0.12° greater in the intervention group (−0.61, 0.85)	41 participants (2 observational studies)	⊕⊕OO Low [43,44], *
right mandibular length	The mean difference in right mandibular length was 1.41 mm greater in the intervention group (0.03, 2.79)	41 participants (2 observational studies)	⊕⊕OO Low [43,44], *
left mandibular length	The mean difference in left mandibular length was 1.47 mm greater in the intervention group (0.16, 2.79)	41 participants (2 observational studies)	⊕⊕OO Low [43,44], *
right mandibular corpus length	The mean difference in right mandibular corpus was 0.25 mm greater in the intervention group (−1.04, 1.53)	41 participants (2 observational studies)	⊕⊕OO Low [43,44], *
left mandibular corpus length	The mean difference in right mandibular corpus was 0.93 mm greater in the intervention group (−0.04, 1.90)	41 participants (2 observational studies)	⊕⊕OO Low [43,44], *
right mandibular ramus height	The mean difference in right mandibular ramus height was 1.61 mm greater in the intervention group (0.43, 2.78)	41 participants (2 observational studies)	⊕⊕OO Low [43,44], *
left mandibular ramus height	The mean difference in left mandibular ramus height was 0.64 mm greater in the intervention group (−0.68, 1.97)	41 participants (2 observational studies)	⊕⊕OO Low [43,44], *
right Co point (AP change)	The mean difference in right Co point was 0.3 mm more anteriorly in the intervention group (−0.86, 1.45)	41 participants (2 observational studies)	⊕OOO Very Low [43,44], *
left Co point (AP change)	The mean difference in left Co point was 0.31 mm more anteriorly in the intervention group (−1.2, 1.81)	41 participants (2 observational studies)	⊕OOO Very Low [43,44], *
right anterior glenoid fossa (AP change)	The mean difference in right anterior glenoid fossa was 0.52 mm more anteriorly in the intervention group (−2.28, 3.32).	41 participants (2 observational studies)	⊕OOO Very Low [43,44], *
left anterior glenoid fossa (AP change)	The mean difference in left anterior glenoid fossa was 0.61 mm more anteriorly in the intervention group (−1.62, 2.83).	41 participants (2 observational studies)	⊕OOO Very Low [43,44], *
right posterior glenoid fossa (AP change)	The mean difference in right posterior glenoid fossa was 0.6 mm more anteriorly in the intervention group (−0.43, 1.62).	41 participants (2 observational studies)	⊕OOO Very Low [43,44], *
left posterior glenoid fossa (AP change)	The mean difference in left posterior glenoid fossa was 0.57 mm in the intervention group (−0.8, 1.93).	41 participants (2 observational studies)	⊕OOO Very Low [43,44], *

* Low quality of evidence (⊕⊕OO) based on the inclusion of observational studies and very low quality (⊕OOO) based on inconsistency according to GRADE criteria, ** outcomes assessed in the RCT started form high quality of evidence (⊕⊕⊕⊕) due to absence of bias and were downgraded if inconsistency, indirectness, imprecision, or publication bias existed, ° = degrees of an angle.

## Data Availability

Not applicable.

## Appendix A

**Table A1:** Search strategy and search string for MEDLINE.

#1 Forsus	127
#2 Forsus Fatigue Resistant Device	75
#3 Herbst	5224
#4 Herbst Appliance	3677
#5 Functional appliance	249,472
#6 Class II appliance	6537
#7 #1 OR#2 OR#3 OR#4 OR#5 OR#6	257,867
#8 Malocclusion, Angle Class II [Mesh]	6618
#9 Class II	120,601
#10 Class II malocclusion	8384
#11 Class II jaw relationship	1019
#12 skeletal Class II	3595
#13 dental Class II	8264
#14 Class II subdivision	240
#15 #8 OR#9 OR#10 OR#11 OR#12 OR#13 OR#14	121,068
#16 #7 AND#15	5709
PubMed search string: (Forsus OR “Forsus Fatigue Resistant Device” OR Herbst OR “Herbst Appliance” “Functional appliance” OR “Class II appliance”) AND ((“Malocclusion, Angle Class II” [Mesh]) OR “Class II” OR “Class II malocclusion” OR “Class II jaw relationship” OR “skeletal Class II” OR “dental Class II” OR “Class II subdivision”)	

**Table A2:** Search strategy and search string for SCOPUS.

#1 Forsus	424
#2 “Forsus Fatigue Resistant Device”	30
#3 Herbst	263,257
#4 “Herbst Appliance”	1562
#5 “Functional appliance”	3101
#6 “Class II appliance”	16
#7 (Forsus OR "Forsus Fatigue Resistant Device" OR Herbst OR "Herbst Appliance" "Functional appliance" OR "Class II appliance")	1160
#8 INDEXTERMS("Malocclusion, Angle Class II")	5765
#9 “Class II”	292,108
#10 “Class II malocclusion”	5832
#11 “Class II jaw relationship”	15
#12 “skeletal Class II”	2570
#13 “dental Class II”	152
#14 “Class II subdivision”	391
#15 ((INDEXTERMS ("Malocclusion, Angle Class II")) OR "Class II" OR "Class II malocclusion" OR "Class II jaw relationship" OR "skeletal Class II" OR "dental Class II" OR "Class II subdivision")	292,108
#16 (Forsus OR "Forsus Fatigue Resistant Device" OR Herbst OR "Herbst Appliance" "Functional appliance" OR "Class II appliance") AND ((INDEXTERMS ("Malocclusion, Angle Class II")) OR "Class II" OR "Class II malocclusion" OR "Class II jaw relationship" OR "skeletal Class II" OR "dental Class II" OR "Class II subdivision")	1017
Scopus search string: (Forsus OR "Forsus Fatigue Resistant Device" OR Herbst OR "Herbst Appliance" "Functional appliance" OR "Class II appliance") AND ((INDEXTERMS ("Malocclusion, Angle Class II")) OR "Class II" OR "Class II malocclusion" OR "Class II jaw relationship" OR "skeletal Class II" OR "dental Class II" OR "Class II subdivision")	

**Table A3:** Search strategy and search string for EMBASE.

#1 Forsus	154
#2 Forsus Fatigue Resistant Device	87
#3 Herbst	5014
#4 Herbst Appliance	430
#5 Functional appliance	802
#6 Class II appliance	4
#7 (Forsus or “Forsus Fatigue Resistant Device” or Herbst or “Herbst Appliance” “Functional appliance” or “Class II appliance”)	5123
#8 exp “Malocclusion, Angle Class II”/	8927
#9 Class II	128,145
#10 Class II malocclusion	2313
#11 Class II jaw relationship	29
#12 skeletal Class II	1221
#13 dental Class II	152
#14 Class II subdivision	119
#15 ((exp “Malocclusion, Angle Class II”/) or “Class II” or “Class II malocclusion” or “Class II jaw relationship” or “skeletal Class II” or “dental Class II” or “Class II subdivision”)	16,777
#16 #7 AND #15	261
Embase search string: (Forsus or “Forsus Fatigue Resistant Device” or Herbst or “Herbst Appliance “Functional appliance” or “Class II appliance”) and ((exp “Malocclusion, Angle Class II”/) or “Class II” or “Class II malocclusion” or “Class II jaw relationship” or “skeletal Class II” or “dental Class II” or1 “Class II subdivision”)	

**Table A4:** Search strategy and search string for CENTRAL.

#1 Forsus	36
#2 Forsus Fatigue Resistant Device	21
#3 Herbst	530
#4 Herbst Appliance	63
#5 Functional appliance	330
#6 Class II appliance	466
#7 #1 OR#2 OR#3 OR#4 OR#5 OR#6	1084
#8 MeSH descriptor: [Malocclusion, Angle Class II] explode all trees	378
#9 Class II	153,189
#10 Class II malocclusion	710
#11 Class II jaw relationship	80
#12 skeletal Class II	1878
#13 dental Class II	3620
#14 Class II subdivision	35
#15 #9 OR #10 OR #11 OR #12 OR #13 OR #14	153,189
#16 #7 AND #15	583
CENTRAL search string: (Forsus OR “Forsus Fatigue Resistant Device” OR Herbst OR “Herbst Appliance” “Functional appliance” OR “Class II appliance”) AND (([mh “Malocclusion, Angle Class II”]) OR “Class II” OR “Class II malocclusion” OR “Class II jaw relationship” OR “skeletal Class II” OR “dental Class II” OR “Class II subdivision”)	

**Table A5:** Search strategy for Cochrane Oral Health Group’s Trial Register.

#1 Forsus	0
#2 Forsus Fatigue Resistant Device	0
#3 Herbst	1
#4 Herbst Appliance	1
#5 Functional appliance	8
#6 Class II appliance	7
#7 Class II	23
#8 Class II malocclusion	9
#9 Class II jaw relationship	1
#10 skeletal Class II	0
#11 dental Class II	16
#12 Class II subdivision	0
#13 Herbst Appliance or Forsus AND Class II malocclusion	0

**Table A6:** Search strategy for ClinicalTrials.gov.

#1 Forsus	5
#2 Forsus Fatigue Resistant Device	3
#3 Herbst	16
#4 Herbst Appliance	14
#5 Functional appliance	41
#6 Class II appliance	47
#7 (Forsus) OR (Forsus Fatigue Resistant Device) OR (Herbst) OR (Herbst Appliance) OR (Functional appliance) OR (Class II appliance)	70
#8 Class II	235
#9 Class II Malocclusion	90
#10 Class II jaw relationship	1
#11 skeletal Class II	27
#12 dental Class II	37
#13 Class II subdivision	0
#14 (Class II) OR (Class II Malocclusion) OR (Class II jaw relationship) OR (skeletal Class II) OR (dental Class II) OR (Class II subdivision)	235
#15 Condition or Disease: (Class II) OR (Class II Malocclusion) OR (Class II jaw relationship) OR (skeletal Class II) OR (dental Class II) OR (Class II subdivision) Intervention/treatment: (Forsus) OR (Forsus Fatigue Resistant Device) OR (Herbst) OR (Herbst Appliance) OR (Functional appliance) OR (Class II appliance)	48

**Table A7:** Search strategy for Google Scholar.

Herbst OR Forsus AND elastics	18,200

**Table A8:** Screenatron Systematic Review Accelerator (advanced search).

(((Forsus OR “Forsus Fatigue Resistant Device” OR Herbst OR “Herbst Appliance” “Functional appliance” OR “Class II appliance”) AND (“Class II” OR “Class II malocclusion” OR “Class II jaw relationship” OR “skeletal Class II” OR “dental Class II” OR “Class II subdivision”)) OR (elastics)	690
